# Psychometric properties of the perceived stress scale in Ethiopian university students

**DOI:** 10.1186/s12889-018-6310-z

**Published:** 2019-01-09

**Authors:** Md Dilshad Manzar, Mohammed Salahuddin, Sony Peter, Ahmad Alghadir, Shahnawaz Anwer, Ahmed S. Bahammam, Seithikurippu R. Pandi-Perumal

**Affiliations:** 1 Department of Nursing, College of Applied Medical Sciences, Majmaah University, Al Majmaah, 11952 Saudi Arabia; 2 Department of Pharmacy, College of Medicine and Health Sciences, Mizan-Tepi University (Mizan Campus), Mizan-Aman, Ethiopia; 3 Department of Biomedical Sciences, College of Medicine and Health Sciences, Mizan-Tepi University (Mizan Campus), Mizan-Aman, Teppi, Ethiopia; 4 Rehabilitation Research Chair, College of Applied Medical Sciences, King Saud University, Riyadh, Saudi Arabia; 5 The University Sleep Disorders Center, College of Medicine, King Saud University, Box 225503, Riyadh, 11324 Saudi Arabia; 6 National Plan for Science and Technology, College of Medicine, King Saud University, Riyadh, Saudi Arabia; 7 Somnogen Canada Inc, College Street, Toronto, ON Canada

**Keywords:** PSS, Anxiety, Stress, Africa, Factor analysis,, McDonald’s omega

## Abstract

**Background:**

Stress is a common psychological condition usually associated with many psycho-physical disorders. Stress and its risk factors are frequently seen in Ethiopians including university students. In such circumstances, a valid measure to screen for stress in Ethiopians is necessary. Therefore, we assessed the psychometric properties of the Perceived Stress Scale (PSS) in Ethiopian university students.

**Methods:**

A cross-sectional study with a simple random sampling method was performed on students of Mizan-Tepi University, Mizan-Aman, Ethiopia. The study presents a psychometric investigation on a sample of 387 students (age = 21.8 ± 3.8 years, and body mass index = 20.8 ± 3.2 kg/m^2^) who completed PSS, Generalized anxiety disorder-7 scale (GAD-7), and a socio-demographics tool. McDonald’s Omega (internal consistency), factor validity for ordinal data and convergent validity (Spearman’s correlation) were assessed.

**Results:**

No ceiling/floor effect was seen for the total or factor scores of the PSS-10 and PSS-4. Two factor model of the PSS-10 was favored by fit indices with Comparative Fit Index> 0.95, Weighted root mean square residual<.05 and root mean square error of approximation<.08. McDonald’s Omega was 0.78 and 0.68 for the PSS-10: Factor-1 and PSS-10: Factor-2, respectively. McDonald’s Omega was 0.70 and 0.54 for the PSS-4: Factor-1 and PSS-4: Factor-2, respectively. There were moderate-strong correlations (*r* = 0.62–0.83) between PSS factors and respective items loading on them. PSS scores were correlated with GAD-7 (*r* = .27–.40, *p* < .01).

**Conclusion:**

The psychometric measures support the validity of the PSS-10 in Ethiopian university students.

**Electronic supplementary material:**

The online version of this article (10.1186/s12889-018-6310-z) contains supplementary material, which is available to authorized users.

## Background

Stress is the reaction when human perceives a discrepancy in his resources and/or the ability to respond to an event or stimulus or stressor [1]. Eventually, stress has been conceptualized into three perspectives: (i) biological, physiology of the stress stimulus and response; (ii) environmental, related to life events; and (iii) psychological, assessment of subjective stress and dealing methods [1, 2]. Psychological stress is associated with asthma, upper respiratory tract infections, smoking, depression, diabetes, epilepsy, HIV/AIDS, herpes viral infections, autoimmune diseases, wound healing, self-reported measures of health behavior and help-seeking [3–5].

Perceived stress is a risk factor for poor sleep quality in Ethiopian university students [6]. Stress is common in various sections of the Ethiopian population such as university students [6], epilepsy patients [7], HIV-infected patients [8], nurses [9], and females students with childhood sexual abuse [10]. Moreover, many risk factors, i.e., substance use [11–13], sleep problems [6, 11, 12], HIV [14], food insecurity [15], poverty [15], and risky sexual behavior [14] for stress and related mental problems are commonly prevalent in Ethiopian populations. It can therefore arguably be considered that there is a prospect of the undiagnosed and under-recognized magnitude of stress in the Ethiopian population. Indeed, similar to most places, depression seems to be more researched in Ethiopia than stress and other related psychological problems. The preponderance of depression in Ethiopian psychometric research is evidenced by the availability of validated tools to measure depression in Ethiopians [16–19]. However, no tool has been validated in Ethiopians to assess stress.

Therefore, in this study, we examined the psychometric validation of one of the most widely used questionnaire tools to evaluate psychological stress, i.e., the Perceived Stress Scale [2, 20]. This tool measures the extent and/or severity of self-reported appraisal of the stressors effect on respondent’s life [20]. There are three versions; 14-item scale called PSS-14, 10-item scale in short PSS-10, and 4-item scale called PSS-4 [3, 20]. The psychometric properties of the PSS have been assessed in various cultures across the globe, but have never been validated in Ethiopians. PSS has been translated into many languages and has been found to have adequate validity and reliability in various demographics of the population [2, 20]. The convergent validity of the PSS has been evaluated by assessing correlation of the PSS scores with the measures of anxiety including the Generalized anxiety disorder-7 (GAD-7) scale and the Hospital Anxiety and Depression Scale (HADS) [2, 21]. However, research is required to ascertain some aspects of the psychometric properties of the PSS. The items of the PSS are ordinal in nature; therefore, it would be better to investigate internal consistency using ordinal alpha or McDonald’s Omega and factorial validity employing polychoric correlation matrix with estimation method suitable for ordinal data like robust diagonally weighted least squares (robust DWLS). However, most of the studies investigating the psychometric properties of the PSS employed the Cronbach’s alpha [2]. Similarly, statistical discrepancies are evident in previous works like use of maximum likelihood estimation for factor analysis, which is more suitable for normally distributed continuous data [22, 23]. Furthermore, the psychometric properties of the PSS-4 are not well studied in the student population. Therefore, this study assessed the psychometric properties, i.e., ceiling/floor effect, factorial validity, internal consistency, item discrimination, and criterion validity like convergent validity, of PSS-10 and PSS-4 in Ethiopian university students. PSS-10 and PSS-4 are both brief measures but PSS-4 is still shorter. If both PSS-10 and PSS-4 show similar psychometric properties then the use of PSS-4 may be favorable.

## Material and methods

### Participants

**Fig. 1:**
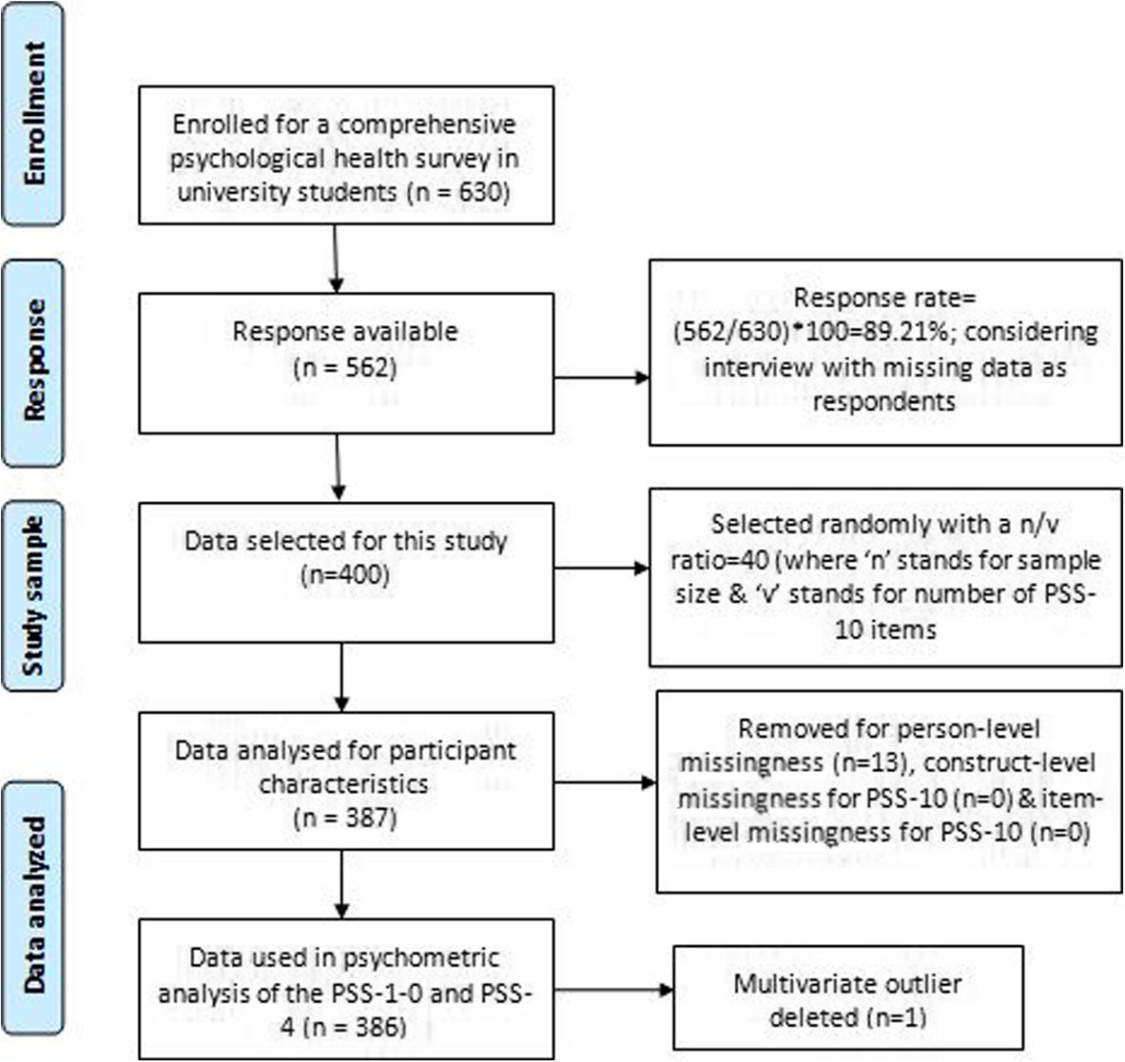
Schematic of study sample

Six hundred and thirty students were initially enrolled from Mizan-Tepi University, Mizan-Aman, Ethiopia for a psychological health survey (Fig. 1). Of these, 562 participated with a response rate of 89.21%. From this, a sample of 400 students was randomly selected for this study (Fig. 1). Here we report the findings from a sample comprising of 387 students (age = 21.8 ± 3.8 years, and body mass index = 20.8 ± 3.2 kg/m^2^) after removing person-level missing values (*n* = 13). There were no construct-level or item-level missing values for PSS scores (Fig. 1). Psychometric properties were investigated in a sample (*n* = 386) after deleting multivariate outlier (*n* = 1) as determined by Mahalanobis distance (χ^2^ = 29.59, df(10), *p* < 0.001) (Fig. 1).

### Procedure

The study was approved by the institutional Ethical committee, College of Medicine and Health Sciences, Mizan-Tepi University, Ethiopia. This was a cross-sectional study with a simple random sampling design. All the participants were informed about the aim and the procedures of the study. Those with self-reported problems of memory and/or use of neuro-psychotic medications were excluded. The participants provided written informed consent. PSS-10, PSS-4, Generalized anxiety disorder-7 scale (GAD-7), and a socio-demographics questionnaire were employed. Ethiopia is home to about eighty languages and students in the university belong to various linguistic ethnicities. Therefore, the study employed English versions of all the questionnaires.

## Measures

### Perceived stress scale

The PSS is a 10-item questionnaire to measure the self-reported level of stress in the respondents by assessing feelings and thoughts during the last month. Each item is scored from 0 (never) to 5 (very often) with a range of 0 to 40 for the total score of the scale. A higher level of stress is indicated by higher scores on this scale [20]. Six items of the PSS-10 measure stress and 4 items measure coping strategy to stress. The PSS-4 is a brief tool derived from the PSS-10 with 4 items, of which 2 items assess stress while 2 items measure coping strategy to stress [20].

### Generalized anxiety disorder – 7 scale

The GAD-7 scale is a 7-item questionnaire to measure the self-reported level of anxiety in the respondents during the preceding two weeks. Each item is scored from 0 (not at all) to 4 (nearly every day). The scores of all items are added to get the total scores, with a range of 0 to 21. The items were designed to quantify symptoms of anxiety according to the Diagnostic and Statistical Manual of Mental Disorders-IV-TR [24]. The GAD-7 has adequate psychometric validity, i.e., convergent validity, diagnostic validity, factorial validity, internal consistency, test-re-test reliability in various populations [25].

### Statistical analysis

SPSS 23.0 with AMOS; which is an add-on module for SPSS along with a plug-in for AMOS and FACTOR version 10.8.04 were used for statistical analysis. Socio-demographics and item analysis were performed by descriptive statistics like frequency, mean with standard deviation, percentage, and Spearman’s correlation between PSS Factor scores and item scores loading on them. Internal consistency was assessed by the McDonald’s Omega. Item discrimination and convergent validity were evaluated by Spearman’s correlation test.

Bartlett’s test of Sphericity, communality, determinant and Kaiser-Meyer-Olkin test of sampling adequacy (KMO) were employed to assess sample size adequacy and sample suitability for factor analysis. Factor analysis was performed using FACTOR version 10.8.04 employing polychoric correlation matrix. Confirmatory factor analysis (CFA) was performed using the robust DWLS extraction with Promin rotation using bootstrap on previously validated models, i.e., both 2-Factor and 1-Factor models for both PSS-10 and PSS-4 [2]. CFA was run on 4 models, i.e., 2 of PSS-10; 1-Factor model (Model-A), and 2-Factor model (Model-B) [22] and 2 models of PSS-4; 1-Factor model (Model-C) and 2-Factor model (Model-D). Multiple indices from different classes of fit measurements were employed [26]. Model fit was indicated by a robust mean and variance-adjusted Chi Square statistics (non-significant *p* value), comparative Fit Index (CFI > 0.95), Weighted root mean square residual (WRMR< 0.5) and root mean square error of approximation (RMSEA< 0.8) [27].

## Results

### Descriptive

More than one third of the students reported no athletic activity. About 26% of students were either under-weight, over-weight or obese. ‘B’ was the most commonly reported grade at the last examination. Almost half of the students practiced Orthodox denomination of the Christianity. About one third of the students did not prefer to report their family’s monthly income (Table 1). The average scores for the PSS and GAD-7 scale are shown in Table 1. Nearly 5% of students reported the use of khat or alcohol (Table 1).

**Table 1:** Socio-demographics of Ethiopian university students

Characteristics	Mean ± SD/ Frequency (Percentage)
Age (yr)	21.8 ± 3.8
Attendance (%)	
Up to 80	49(12.7%)
80–90	39(10.1%)
90–100	299(77.3%)
Athletic activity (min/day)	
No activity	145(37.5%)
Less than 60	51(13.2%)
60–120	151(39%)
More than 120	40(10.3%)
BMI (Kg/m^2^)	
Underweight	66(17.1%)
Normal	286(73.9%)
Overweight	27(7.0%)
Obese	8(2.1%)
Gender	
Male	321(82.9%)
Female	66(17.1%)
Grade point average (at last semester examination)	
C	94(24.3%)
C+	43(11.1%)
B-	24(6.2%)
B	107(27.6%)
B+	58(15.0%)
A-	52(13.4%)
A/A+	5(1.3%)
Un-reported	4(1.0%)
Religion	
Christianity	
Catholic	1(0.3%)
Orthodox	187(48.3%)
Protestants	122(31.5%)
Islam	68(17.8%)
Others	9(2.3%)
Monthly Family Income (In Birr)	
Very Low (less than 445)	41(10.6%)
Low (446–1200)	67(17.3%)
Average (1201–2500)	53(13.7%)
Above average (2501–3500)	30(7.8%)
High (greater than 3500)	72(18.6%)
Unknown	124(32.0%)
GAD-7 scale	7.2 ± 4.9
PSS	
PSS-10:Factor-1	10.72 ± 4.74
PSS-10:Factor-2	7.35 ± 3.25
PSS-10:total	18.07 ± 4.72
PSS-4: total	7.14 ± 2.43
Substance use	
Alcohol	
No	364(94.1%)
Yes	23(5.9%)
Chat Chewing	
No	370(95.6%)
Yes	17(4.4%)
Cigarette	
No	385(99.5)
Yes	2(0.5%)

SD: Standard Deviation; BMI: Body mass index

GAD-7: Generalized Anxiety Disorder-7 scale

PSS: Perceived stress scale

### Preliminary item analysis

Item analysis for the PSS scores in the study participants is shown in Table 2. According to previous work, we scored ceiling or floor effect if more than 15% of respondents reported the highest or the lowest score, respectively [11, 28]. Item-1, item-2, item-3, item-4, item-6, item-9 and item-10 showed floor effect; while none had ceiling effect (Table 2). However, there was no issue of ceiling/floor effect for PSS-10 total, PSS-10: Factor-1, PSS-10: Factor-2, PSS-4 total, PSS-4: Factor-1 and PSS-4: Factor-2 scores (Table 2).

**Table 2:** Descriptive statistics, item-Factor correlations, and Communality of the Perceived Stress Scale (PSS-10 & PSS-4) in Ethiopian university students

Perceived Stress Scale items	Item-Factor score Correlation^#^				Communality		Percentage distribution of Item scores				
	PSS-10		PSS-4		PSS-10	PSS-4	0	1	2	3	4
	Factor-1	Factor-2	Factor-1	Factor-2							
Item-1	.66^*^				.41		21.0	17.9	40.2	12.7	8.3
Item-2	.70^*^		.83^*^		.48	.35	16.8	20.5	37.0	15.8	9.8
Item-3	.65^*^				.37		19.4	18.7	36.3	17.4	8.3
Item-4		.68^*^		.83^*^	.26	.08	20.2	29.0	26.2	11.9	12.7
Item-5		.67^*^		.75^*^	.38	.20	9.1	25.6	38.1	16.6	10.6
Item-6	.62^*^				.32		16.1	22.8	32.1	22.5	6.5
Item-7		.69^*^			.45		14.2	31.1	32.1	13.0	9.6
Item-8		.67^*^			.35		9.8	23.1	35.0	20.5	11.7
Item-9	.68^*^				.44		16.1	15.0	39.1	19.2	10.6
Item-10	.66^*^		.80^*^		.42	.36	15.8	27.5	33.7	16.3	6.7

^#^Spearman’s correlation coefficient

^*^p < 0.01

Items of PSS-10: Item-1 to Item-10

Items of PSS-4: items-2, 4, 5 and 10

### Factorial validity

#### Sample adequacy and suitability for factor analysis

The Kaiser-Meyer-Olkin Test of Sampling Adequacy (KMO) values were 0.83 and 0.54, respectively for the PSS-10 and the PSS-4, which showed that the degree of common variance among PSS item scores was meritorious for the PSS-10 but poor for PSS-4 [29]. The Bartlett’s test (<.001) imply that the original matrix is not an identity matrix, i.e., there is no problem of singularity in the measured variables-PSS item scores for both the PSS-10 and the PSS-4 [29]. The determinant score; PSS-10 (0.14) and PSS-4 (0.74), found that there was no issue of multi-collinearity in the PSS item scores [29]. The communality for all the PSS-10 item scores was above 0.2 (Table 2, Additional file 1), implying that proportion of variance explained by the common factors was adequate [30]. However, for the PSS-4, one of the items had a communality of 0.08 ( Table 2), suggesting that the proportion of variance explained by the common factors was not adequate for the PSS-4 [30].

#### Confirmatory factor analysis

**Fig. 2:**
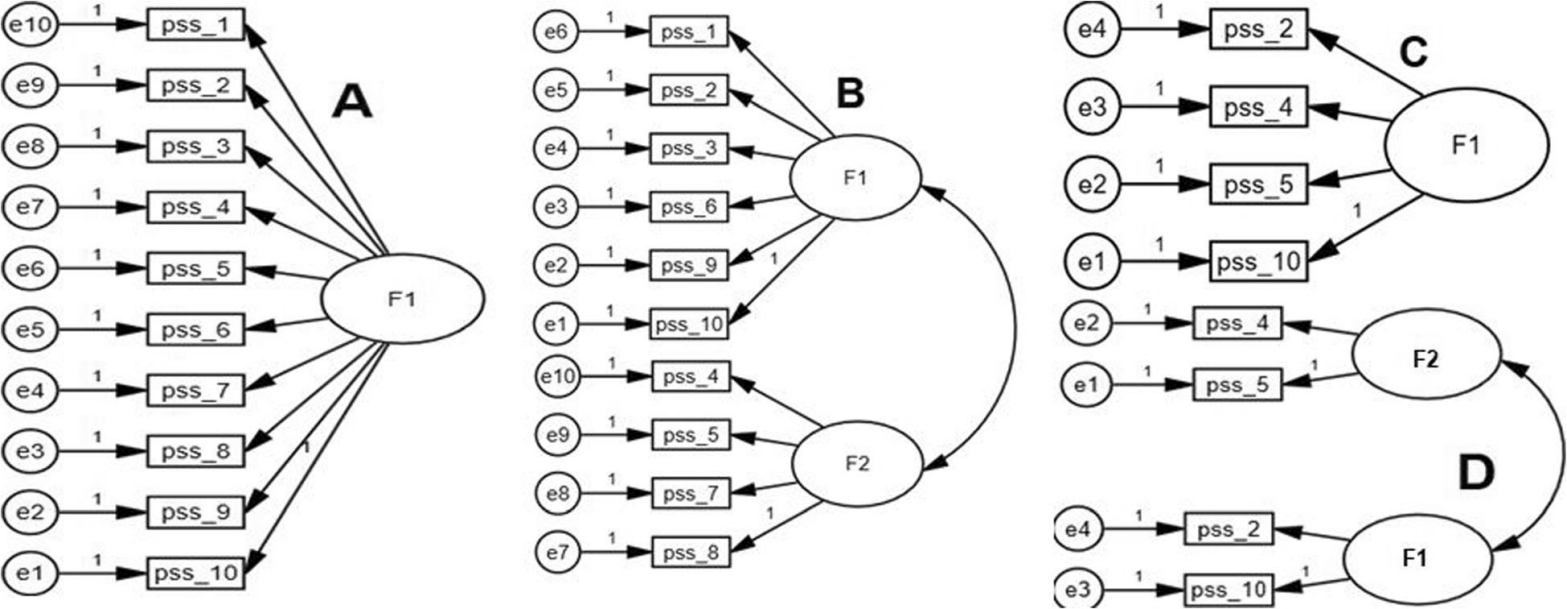
Confirmatory factor analysis models of the Perceived Stress Scale (PSS-10 & PSS-4) in Ethiopian university students. PSS-10 models. A: 1-Factor model, B: 2-Factor model PSS-4 models. Model-C: 1-Factor model, Model-D: 2-Factor model pss_1 to pss_10: items of the PSS-10, PSS-4 comprise of pss_2, pss_4, pss_5 and pss_10. All coefficients are standardized. *Ovals* latent variables, *rectangles* measured variables, *circles* error terms, *single-headed arrows* between *ovals* and *rectangles* factor loadings, *single-headed arrows* between *circles* and *rectangles* error terms

PSS-10: model-B (Fig. 2) showed the best fit; highest value of the CFI (.989), lowest value of the RMSEA (.038) and optimum value of WRMR (.034) (Additional file 2, Table 3). Though, model-D, showed favorable values of fit indices but one of its items had very low communality (Fig. 2, Table 2-3).

**Table 3:** Fit statistics of the Perceived Stress Scale (PSS-10 & PSS-4) in Ethiopian university students

Models	CFI	WRMR	RMSEA	χ^2#^	df	*p*	χ^2^/df
PSS-10							
A	.891	.088	.102 (.070–.123)	175.459	35	<.001	5.014
B	.989	.034	.038 (.017–.046)	40.235	26	.037	1.548
PSS-4							
C	.723	.100	.198(.122–.285)	32.317	2	<.001	16.158
D	.976	.000	.083(.000–.182)	3.680	1	.055	3.680

^#^: Robust Mean and Variance-Adjusted Chi Square

CFI: Comparative Fit Index, WRMR: Weighted root mean square residual, RMSEA: root mean square error of approximation

PSS-10 models; A: 1-F model, B: 2-F model

PSS-4 models; C: 1-F model, D: 2-F model

### Internal consistency and item discrimination

McDonald’s Omega for the PSS-10: Factor-1 and the PSS-10: Factor-2 was 0.80 and 0.68, respectively While, McDonald’s Omega was 0.70 and 0.54 for the PSS-4: Factor-1 and the PSS-4: Factor-2, respectively. The item-Factor correlations for the PSS-10: Factor-1 (*r* = 0.62–0.70, *p* < .01) and the PSS-10: Factor-2 (*r* = 0.67–0.69, *p* < .01) were moderate to strong (Table 2). The item-Factor correlations for the PSS-4: Factor-1 (*r* = 0.80–0.83, *p* < .01) and the PSS-4: Factor-2 (*r* = 0.75–0.83, p < .01) were strong (Table 2).

### Convergent validity: Correlation between GAD-7 and PSS scores

The PSS-10 total score and the PSS-10: Factor-1 scores had a weak to moderate correlation (*r* = 0.34 and *r* = 0.40, *p* < .01 respectively) with the GAD-7 total score (Table 4). The PSS-4 total and the PSS-4: Factor-1 scores had a weak to moderate correlation (*r* = 0.27 and *r* = 0.38, p < .01, respectively) with the GAD-7 total score (Table 4).

**Table 4:** Criterion validity: Correlation of the Perceived Stress Scale with Generalized Anxiety Disorder-7 scale in Ethiopian university students

PSS scores	GAD-7 total
PSS-10	
Factor-1	.40^**^
Factor-2	−.05
PSS total	.35^**^
PSS-4	
Factor-1	.38^**^
Factor-2	−.01
PSS total	.27^**^

* *p* < 0.05, ** *p* < 0.01

GAD-7: Generalized Anxiety Disorder-7 scale

## Discussion

This is the first study to evaluate the psychometric validation measures of the PSS-10 and the PSS-4 in Ethiopian Africans in general and university students in particular. The investigation found sufficient level of the ceiling/floor effect, item discrimination, internal consistency, convergent validity, and factorial validity for PSS-10 in the study population.

### Preliminary item analysis

The absence of both the floor and ceiling effects entails that even at the lowest or the highest scores of the PSS total and factor scores for both the PSS-10 and the PSS-4, the variance of the measurement is not unaccounted [28]. This favors the structural validity of the PSS-10 and the PSS-4 in Ethiopian university students as a self-reported measure of stress. Of the few previous studies that investigated this aspect of the validity of the PSS, Wu and Amtmann reported that there was no major floor and/or ceiling effects in Americans Multiple Sclerosis patients [31].

### Factorial validity

CFA (Fig. 2 and Table 4) favored the 2-Factor model for the PSS-10 in the Ethiopian university students. Most of the previous studies have also favored the 2-Factor model of the PSS-10 and PSS-4 [2, 21, 32, 33]. However, some of the studies reported a bi-factor model [34] while some reported a 1-Factor model [2]. Similarly, previous works have validated a 2-Factor model for the PSS-10 among Americans, Thai and Turkish university students [2, 35]. Incidentally, the 2-Factor structures of both the PSS-10 and the PSS-4 are theoretically favored over a unidimensional model because some of the items measure stress, while others assesses the coping strategy to the stress [20].

### Internal consistency and item discrimination

The internal consistency as assessed by the McDonald’s Omega value for one of the factors of the PSS-10 was slightly lower than the minimum acceptable value of 0.70 (Additional file 3). However, internal consistency was very poor for the PSS-4: Factor-2. Most of the previous studies have reported the Cronbach’s alpha (0.74 to 0.91) for assessing internal consistency of the PSS, [2]. The item discrimination index, i.e., item-Factor correlations were all above 0.5 for both the PSS-10 as well as the PSS-4. This implies that item scores of the PSS had the ability to distinguish between high and low scoring individuals in the study population [23].

### Criterion-related validity: Convergent validity

Stress conditions are closely associated with anxiety but they represent different psychological constructs [36], therefore previous studies have investigated the relationship between the PSS and measures of anxiety including the GAD-7 to establish its criterion validity [2]. However, noticeably this difference in the construct is perhaps accountable for the moderate level of correlation between the PSS and the GAD-7 scores in this study population of Ethiopian university students. Nevertheless, it can reasonably be concluded that the correlation between the PSS scores, i.e., PSS-10 total, PSS-4 total, PSS-10: Factor-1 and PSS-4: Factor-1 scores with the GAD-7 (Additional file 4, Table 4) favors the convergent validity of the PSS-10 and the PSS-4 in this population of Ethiopian university students. Previous studies have also supported the convergent validity of the PSS in different populations by assessing its correlation with measures of anxiety [21, 32–34]. Maroufizadeh et al. 2014 found a moderate association between the PSS and anxiety subscale of the Depression Anxiety Stress-21 scale in Persian asthmatic adults [33]. The convergent validity of the PSS had been supported by assessing correlations employing measures of anxiety-such as Spielberger Trait Anxiety Inventory in Spanish Americans [34], Hospital Anxiety and Depression Scale among American patients with systemic lupus erythematosus [21], and the GAD-7 among community-dwelling Hispanic Americans [32]. PSS Factor-2 did not correlate with these measures (Table 4) because it does not assess stress but coping strategy to stress conditions [20].

In summary, PSS-10 was found to have no major issues of the ceiling/floor effect, favorable factorial validity for 2-Factor model, internal consistency, item discrimination, and convergent validity among Ethiopian university students. However, psychometric properties were not adequate for the PSS-4 in the study population.

Limitations of the current study include a small number of female student’s participants, non-assessment of diagnostic validity, and test-re-test reliability. The questionnaire was not administered in the first language of the respondents which may have limitations, but it is worth mentioning that the medium of instruction is English in the Ethiopian universities. The lower response rate among female participants was one of the important reasons that led to bias in gender representation in the final sample. Future works employing diagnostic clinical interview to explore the concurrent validity of the PSS in Ethiopians are needed. Nevertheless, there are notable merits of this study. We found adequate psychometric validation for the PSS-10 in a population which has limited access to expert medicine professionals and facilities. Many risk factors for behavioral problems are prevalent in Ethiopia. Therefore, the availability of a validated measure of stress is very important. The psychometric properties assessed in this study do support application of the PSS-10 to screen stress among Ethiopians.

## Conclusion

The study provides support for the psychometric validation of the PSS-10 in the Ethiopian university students.

## Additional files

Additional file 1: Item-Factor correlations, and Communality of the Perceived Stress Scale (PSS 10) in Ethiopian university students. Highlighted values: total survey sample (*n* = 562). Non-highlighted values: study sample (*n* = 386). (DOCX 15 kb)

Additional file 2: Fit statistics of the Perceived Stress Scale (PSS-10) in Ethiopian university students. Highlighted values: total survey sample (n = 562). Non-highlighted values: study sample (n = 386). (DOCX 13 kb)

Additional file 3: McDonald’s Omega of the 2-Factor model of the PSS-10 in Ethiopian university students. Highlighted values: total survey sample (n = 562). Non-highlighted values: study sample (n = 386) (DOCX 11 kb)

Additional file 4: Convergent validity of the PSS-10 with Generalized Anxiety Disorder-7 scale in Ethiopian university students. Highlighted values: total survey sample (n = 562). Non-highlighted values: study sample (*n* = 386). (DOCX 12 kb)

Additional file 5: The file contains the data used in the psychometric analysis of the Perceived stress scale (PSS) in Ethiopian university students. The data file contain data related to participants’ age, body mass index, athletic activity, grade at last examination, religion, gender, attendance, substance use(alcohol, smoking and khat use), PSS scores, generalized anxiety scale-7 total score. (DAT 25 kb)

## Abbreviations

CFA: confirmatory factor analysis
CFI: Comparative Fit Index
DWLS: diagonally weighted least squares
GAD: Generalized anxiety disorder
KMO: Kaiser-Meyer-Olkin Test of Sampling Adequacy
PSS: Perceived stress scale
RMSEA: root mean square error of approximation
WRMR: weighted root mean square residual
