# Supplementary material for: Psychometric properties of the perceived stress scale in Ethiopian university students
Source: BMC Public Health. 2019 Jan 9;19:41. doi: 10.1186/s12889-018-6310-z (PMC6325789; doi:10.1186/s12889-018-6310-z)
Supplement: Supplementary file 1 — Item-Factor correlations, and Communality of the Perceived Stress Scale (PSS 10) in Ethiopian university students. Highlighted values: total survey sample (n = 562). Non-highlighted values: study sample (n = 386). (DOCX 15 kb) [file 12889_2018_6310_MOESM1_ESM.docx]

Item-Factor correlations, and Communality of the Perceived Stress Scale (PSS-10) in Ethiopian university students

| Perceived Stress  Scale items | Item-Factor score Correlation^#^ | | Communality |
| --- | --- | --- | --- |
|  | PSS-10 | | PSS-10 |
|  | Factor-1 | Factor-2 |  |
| Item-1 | .66^*^ / .67^*^ |  | .41/.39 |
| Item-2 | .70^*^ /.69^*^ |  | .48/.48 |
| Item-3 | .65^*^ / .66^*^ |  | .37/.36 |
| Item-4 |  | .68^*^ /.67^*^ | .26/.26 |
| Item-5 |  | .67^*^ /.68^*^ | .38/.38 |
| Item-6 | .62^*^ /.60^*^ |  | .32/.32 |
| Item-7 |  | .69^*^ /.67^*^ | .45/.44 |
| Item-8 |  | .67^*^ / .67^*^ | .35/.36 |
| Item-9 | .68^*^ /.67^*^ |  | .44/.44 |
| Item-10 | .66^*^ / .67^*^ |  | .42/.41 |

^#^ Spearman’s correlation coefficient

^*^ *p* < 0.01

Items of PSS-10: Item-1 to Item-10

Highlighted values: total survey sample (n=562)

Non-highlighted values: study sample (n=386)
