# Supplementary material for: Psychometric properties of the perceived stress scale in Ethiopian university students
Source: BMC Public Health. 2019 Jan 9;19:41. doi: 10.1186/s12889-018-6310-z (PMC6325789; doi:10.1186/s12889-018-6310-z)
Supplement: Supplementary file 2 — Fit statistics of the Perceived Stress Scale (PSS-10) in Ethiopian university students. Highlighted values: total survey sample (n = 562). Non-highlighted values: study sample (n = 386). (DOCX 13 kb) [file 12889_2018_6310_MOESM2_ESM.docx]

Fit statistics of the Perceived Stress Scale (PSS-10) in Ethiopian university students

| Models | CFI | WRMR | RMSEA | χ^2#^ | df | *p* | χ^2^/df |
| --- | --- | --- | --- | --- | --- | --- | --- |
| PSS-10 | | | | | | | |
| A | .891/.888 | .088/.088 | .102 (.070-.123)/ .104 (.063-.123) | 175.459/180.456 | 35/35 | <.001/<.001 | 5.014/5.156 |
| B | .989/.992 | .034/.035 | .038 (.017-.046)/ .034 (.011-.039) | 40.235/37.310 | 26/26 | .037/.069 | 1.548/1.435 |

^#^: Robust Mean and Variance-Adjusted Chi Square

CFI: Comparative Fit Index, WRMR: Weighted root mean square residual, RMSEA: root mean square error of approximation

PSS-10 models; A: 1-F model, B: 2-F model

Highlighted values: total survey sample (n=562)

Non-highlighted values: study sample (n=386)
