# Supplementary material for: Psychometric properties of the perceived stress scale in Ethiopian university students
Source: BMC Public Health. 2019 Jan 9;19:41. doi: 10.1186/s12889-018-6310-z (PMC6325789; doi:10.1186/s12889-018-6310-z)
Supplement: Supplementary file 3 — McDonald’s Omega of the 2-Factor model of the PSS-10 in Ethiopian university students. Highlighted values: total survey sample (n = 562). Non-highlighted values: study sample (n = 386) (DOCX 11 kb) [file 12889_2018_6310_MOESM3_ESM.docx]

McDonald’s Omega of the 2-Factor model of the PSS-10 in Ethiopian university students

|  | McDonald’s  Omega |
| --- | --- |
| PSS-10 |  |
| Factor-1 | 0.78/.79 |
| Factor-2 | 0.68/.67 |

Highlighted values: total survey sample (n=562)

Non-highlighted text: study sample (n=386)
