# Supplementary material for: Psychometric properties of the perceived stress scale in Ethiopian university students
Source: BMC Public Health. 2019 Jan 9;19:41. doi: 10.1186/s12889-018-6310-z (PMC6325789; doi:10.1186/s12889-018-6310-z)
Supplement: Supplementary file 4 — Convergent validity of the PSS-10 with Generalized Anxiety Disorder-7 scale in Ethiopian university students. Highlighted values: total survey sample (n = 562). Non-highlighted values: study sample (n = 386). (DOCX 12 kb) [file 12889_2018_6310_MOESM4_ESM.docx]

Convergent validity of the PSS-10 with Generalized Anxiety Disorder-7 scale in Ethiopian university students

| PSS scores | GAD-7 total |
| --- | --- |
| PSS-10 |  |
| Factor-1 | .40^**^/.40^**^ |
| Factor-2 | -.05 /.08 |
| PSS total | .35^**^ /.33^**^ |

* *p* < 0.05, ** *p* < 0.01

GAD-7: Generalized Anxiety Disorder-7 scale

Highlighted values: total survey sample (n=562)

Non-highlighted text: study sample (n=386)
